# Ontario Veterinary College

**Published:** 1903-01

**Authors:** 


### COMMENCEMENT EXERCISES.

### ONTARIO VETERINARY COLLEGE.

Christmas examinations of this college were held December 23, 1902, in the college buildings. The usual board of examiners, composed of prominent veterinary surgeons engaged in the active practice of their profession, granted diplomas to the following gentlemen: William G. Chrisman, Harrisonburg, Va.; Peter Crerar, Russell, Manitoba; Fred. J. Delaine, Emerson, Manitoba; Edward L. Fryer, Jr., Blakely, Ga.; Richard L, Kramlich, Fogelsville, Pa.; E. J. Murphy, Metcalfe, Ontario; H. Wynn Nobles, Hastings, Michigan; Matthew S. Suttle, Paterson, New Jersey; William Thompson, Minnedosa, Manitoba; John E. Wurm, Ubley, Michigan.

---

The Attorney-General of Michigan has ruled that the State Veterinary Examining Board must accept diplomas from the Grand Rapids Veterinary College and register under the State law all those who hold the same.

"Parturient Paralysis" is the title of a press bulletin issued by Dr. Chas. F. Dawson, Station Veterinarian of the Florida Experiment Station. Reference is made therein to the value of the potassium iodide treatment.

